# Depressive symptoms measured using the Edinburgh Postnatal Depression Scale in mothers and partners in the ALSPAC Study: A data note

**DOI:** 10.12688/wellcomeopenres.15925.2

**Published:** 2020-08-25

**Authors:** Elise Paul, Rebecca M. Pearson

**Affiliations:** 1 Medical School, University of Bristol, Bristol, UK

**Keywords:** ALSPAC, depression, longitudinal cohort, EPDS, intergenerational, gender

## Abstract

Depression is a leading cause of disability and is associated with a number of adverse offspring outcomes when it occurs in parents. Depression is present in men and women at different rates, and recent research suggests that symptom profiles between the sexes may differ. Longitudinal data are needed to answer remaining questions about the long-term course, gender differences, antecedents and outcomes of depression. The Avon Longitudinal Study of Parents and Children (ALSPAC) is a large birth cohort study in England which administered one of the most commonly used depression instruments, the Edinburgh Postnatal Depression Scale (EPDS) at 11 timepoints in mothers and at 10 timepoints in their partners. In addition to repeated measurements of the EPDS, ALSPAC has a wealth of participant data on biological, social, demographic, and lifestyle factors. The purpose of this data note is to introduce potential users of the data to the characteristics of the EPDS in ALSPAC, as well as some key considerations when using the data.

## Introduction

Depression is one of the common mental disorders and is a leading cause of disability worldwide ^1^. Previous research has also shown that individuals suffering from depression are at increased risk of transmitting this and other mental health problems to their offspring ^2– 4^. Understanding how depression unfolds over time as well as its risk factors in general population samples is therefore an urgent public health priority.

Population wide studies consistently show that depression is more common in women than in men ^5^. Depression in the perinatal period has received an increasing amount of research attention. Research indicates that depression in both mothers and fathers in the time before and shortly after the birth of a child is common ^6^ and is linked with a range of negative offspring outcomes in longitudinal studies ^3, 4, 7– 10^.

The Avon Longitudinal Study of Parents and Children (ALSPAC) is a birth cohort study of over 20,000 women, partners and their children that started in the early 1990s. Data collection is still ongoing and includes a wealth of repeated measurements of personality characteristics, mental health, biological measurements, lifestyle factors and sociodemographic information. One of these repeated measurements is of depressive symptoms via the Edinburgh Postnatal Depression Scale (EPDS) ^11^, the most widely used perinatal depression instrument which has been translated into over 60 languages ^12^. Because the EPDS was originally developed to measure depression in the perinatal period, somatic depression symptoms (e.g. fatigue or changes in appetite) were omitted. Despite its original intended use, the EPDS is also used to assess depressive symptoms outside of the perinatal period ^13– 16^ and is administered to fathers as well ^4, 17^.

Over a dozen published ALSPAC studies have used EPDS data in mothers, partners, or both mothers and partners ^8, 16, 18– 28^. One early finding using ALSPAC data is that depressive symptoms were just as common during pregnancy as in the postnatal period ^29^. Another ALSPAC study found that adolescents of mothers who had been depressed during and shortly after pregnancy were at significant risk for a diagnosis of depression ^10^. More recently it was demonstrated that the prevalence of depression in pregnancy is higher (25%) in the second generation of ALSPAC mothers than in the first (17%) ^30^.

There are still many unanswered questions regarding depression in mothers and partners. The aim of this data note is to describe the longitudinal data on maternal and partner depression available from ALSPAC to facilitate future research on depression.

## Methods

### The ALSPAC sample

The Avon Longitudinal Study of parents and Children (ALSPAC) is a longitudinal birth cohort that recruited pregnant women residing in Avon, UK with expected dates of delivery between 1 April 1991 and 31 December 1992. The initial number of pregnancies was 14,541, which resulted in 14,062 live births and 13,988 children alive by the age of 1 year. Further details on the representativeness, cohort profile and recruitment have been published elsewhere ^31– 33^. The ALSPAC study website contains details of all data available through a fully searchable data dictionary and variable search tool.

Fathers were not initially enrolled in the study directly ^34^. Instead, mothers were sent a questionnaire with the option of passing it on to him to complete. Consequently, no information on the number of mothers who invited their partners to participate is available. At least one questionnaire was returned by 75% of the partners of enrolled women.

Starting in 2014, study data were collected and managed using REDCap (Research Electronic Data Capture) version 7.4.9 hosted at the University of Bristol ^35, 36^. REDCap is a secure, web-based software platform designed to support data capture for research studies.

### Ethical approval and consent

Ethical approval for the study was obtained from the ALSPAC Law and Ethics Committee and the local research ethics committees. Full details of the approvals are available from the study website.

Written informed consent for the use of data collected via questionnaires and clinics was obtained from participants following the recommendations of the ALSPAC Ethics and Law Committee at the time.

### EPDS administration in ALSPAC

The EPDS was administered to ALSPAC mothers and their partners at 11 and 10 timepoints, respectively. Table 1, Table 2 present a complete list of these data collection timepoints which correspond to the focal child’s average age within ALSPAC, as well as the ALSPAC data file and variable names. Nine of the timepoints are the same in both mothers and partners with respect to the focal child’s age: 18 weeks’ gestation, 8 weeks’ post-partum, 8 months, 1 year 9 months, 2 years 9 months, 5 years 1 month, 6 years 1 month, 8 years 1 month, and 11 years 2 months.

**Table 1.** Source of Edinburgh Postnatal Depression Scale (EPDS) item-level data, child age at measurement, and mother variable names in ALSPAC.

Occasion	Child age	Source File in ALSPAC	List of Mother EPDS Variable Names in ALSPAC
1	18 wks. gestation	b_4f	b360 b361 b362 b363 b364 b365 b366 b367 b368 b369
2	32 wks. gestation	c_8a	c590 c591 c592 c593 c594 c595 c596 c597 c598 c599
3	8 wks. post-partum	e_4f	e380 e381 e382 e383 e384 e385 e386 e387 e388 e389
4	8 mos. post-partum	f_2b	f190 f191 f192 f193 f194 f195 f196 f197 f198 f199
5	1 yr. 9 mos. post-partum	g_5c	g280 g281 g282 g283 g284 g285 g286 g287 g288 g289
6	2 yrs. 9 mos. post-partum	h_6d	h190 h191 h192 h193 h194 h195 h196 h197 h198 h199
7	5 yrs. 1 mo. post-partum	k_r1b	k3030 k3031 k3032 k3033 k3034 k3035 k3036 k3037 k3038 k3039
8	6 yrs. 1 mo. post-partum	l_r1b	l2010 l2011 l2012 l2013 l2014 l2015 l2016 l2017 l2018 l2019
9	8 yrs. 1 mo. post-partum	n_3a	n6060 n6061 n6062 n6063 n6064 n6065 n6066 n6067 n6068 n6069
10	11 yrs. 2 mos. post-partum	r_r1b	r4010 r4011 r4012 r4013 r4014 r4015 r4016 r4017 r4018 r4019
11	18 yrs. post-partum	t_2a	t3240 t3241 t3242 t3243 t3244 t3245 t3246 t3247 t3248 t3249

**Table 2.** Source of Edinburgh Postnatal Depression Scale (EPDS) item-level data, child age at measurement, and partner variable names in ALSPAC.

Occasion	Child age	Source File in ALSPAC	List of Partner EPDS Variable Names in ALSPAC
1	18 wks. gestation	pb_4b	pb250, pb251, pb252, pb253, pb254, pb255, pb256, pb257, pb258, pb259
2	8 wks. post-partum	pc_3a	pc092, pc093, pc094, pc095, pc096, pc097, pc098, pc099, pc100, pc101
3	8 mos. post-partum	pd_7b	pd190, pd191, pd192, pd193, pd194, pd195, pd196, pd197, pd198, pd199
4	1 yr. 9 mos. post-partum	pe_4a	pe280, pe281, pe282, pe283, pe284, pe285, pe286, pe287, pe288, pe289
5	2 yrs. 9 mos. post-partum	pf_r1a	pf4030, pf4031, pf4032, pf4033, pf4034, pf4035, pf4036, pf4037, pf4038, pf4039
6	5 yrs. 1 mo. post-partum	ph_1c	ph3030, ph3031, ph3032, ph3033, ph3034, ph3035, ph3036, ph3037, ph3038, ph3039
7	6 yrs. 1 mo. post-partum	pj_r1a	pj2010, pj2011, pj2012, pj2013, pj2014, pj2015, pj2016, pj2017, pj2018, pj2019
8	8 yrs. 1 mo. post-partum	pl_r1b	pl6060, pl6061, pl6062, pl6063, pl6064, pl6065, pl6066, pl6067, pl6068, pl6069
9	11 yrs. 2 mos. post-partum	pp_r1b	pp4010, pp4011, pp4012, pp4013, pp4014, pp4015, pp4016, pp4017, pp4018, pp4019
10	21 yrs. post-partum	fa_1b	fa3240, fa3241, fa3242, fa3243, fa3244, fa3245, fa3246, fa3247, fa3248, fa3249

### Description of the population

Due to attrition and other sources of missing data, sample size of mothers with EPDS data at each timepoint varies, from 12,151 at 18 weeks’ gestation to 4,107 at 18 years post-partum. In partners (all of whom were male), sample sizes vary from 9,846 at 18 weeks’ gestation to 1,951 at 21 years post-partum ( Table 3, Table 4). A total of 14,169 mothers have valid EPDS data for at least one timepoint, with 2,854 of these having EPDS data for all 11 timepoints, and 1,476 mothers have data for zero EPDS assessments. In partners, 14,915 have at least one EPDS assessment, with 4,067 having all 10 assessments and 1,476 having zero. As can be seen in Table 5, Table 6, missingness on EPDS data appears to be related to several demographic characteristics.

**Table 3.** Descriptive statistics and reliability of the Edinburgh Postnatal Depression Scale (EPDS) in mothers in ALSPAC.

Occasion	Mean Maternal Age (SD)	Mean Child Age (SD)	Sample Size	Mean EPDS (SD)	Above EPDS Threshold (≥13)	Above EPDS Threshold (≥10)	α
1	27.76 (4.93)	20.85 (5.66) wks. gest.	12,151	6.99 (4.87)	13.91%	28.17%	0.849
2	28.64 (4.86)	32.65 (1.55) wks. gest.	12,190	7.09 (5.09)	15.23%	29.99%	0.866
3	29.05 (4.82)	9.93 (3.72) wks. post	11,816	6.06 (4.79)	10.16%	21.56%	0.857
4	29.20 (4.77)	8.77 (1.49) mos.	11,318	5.42 (4.70)	8.82%	17.79%	0.867
5	29.97 (4.75)	21.15 (1.18) mos.	10,384	5.72 (4.80)	9.87%	19.98%	0.868
6	32.05 (4.73)	2.81 (0.12) yrs.	9,667	6.29 (5.05)	12.39%	24.11%	0.882
7	34.00 (4.66)	5.13 (0.11) yrs.	8,937	6.05 (5.04)	12.38%	22.66%	0.885
8	35.08 (4.61)	6.17 (0.55) yrs.	8,526	6.35 (5.15)	13.52%	25.63%	0.889
9	37.76 (4.58)	8.26 (0.26) yrs.	7,762	6.14 (5.21)	12.77%	24.05%	0.884
10	40.32 (4.62)	11.28 (0.15) yrs.	7,512	5.81 (5.29)	12.55%	22.64%	0.888
11	48.60 (4.48)	18.47 (0.52) yrs.	4,107	7.47 (5.45)	18.24%	32.36%	0.884

**Table 4.** Descriptive statistics and reliability of the Edinburgh Postnatal Depression Scale (EPDS) in partners in ALSPAC.

Occasion	Mean Partner Age (SD)	Mean Child Age (SD)	Sample Size	Mean EPDS (SD)	Above EPDS Threshold (≥13)	Above EPDS Threshold (≥10)	α
1	30.40 (5.77)	20.23 (3.50) wks. gest.	9,846	4.23 (3.94)	4.08%	10.54%	0.812
2	31.08 (5.73)	10.28 (4.49) wks. post	8,428	3.79 (3.85)	3.60%	9.07%	0.825
3	31.81 (5.53)	8.66 (1.59) mos.	7,165	3.36 (3.70)	2.96%	7.31%	0.823
4	33.24 (5.02)	21.45 (1.22) mos.	6,167	3.66 (3.82)	3.50%	8.46%	0.840
5	34.30 (5.62)	2.85 (0.17) yrs.	5,387	3.79 (3.88)	3.81%	9.17%	0.833
6	36.62	5.17 (0.11) yrs.	4,496	3.95 (4.01)	4.34%	10.36%	0.852
7	37.86 (5.62)	6.18 (0.17) yrs.	4,449	4.46 (4.37)	6.32%	13.98%	0.863
8	39.94	8.26 (0.30) yrs.	3,939	4.27 (4.41)	6.12%	12.77%	0.866
9	43.24 (5.54)	11.28 (0.15) yrs.	3,586	3.93 (4.35)	5.52%	11.85%	0.859
10	53.34 (5.40)	20.24 (0.63) yrs.	1,951	5.85 (4.69)	9.17%	20.30%	0.856

### Description of the population

The EPDS was administered to ALSPAC mothers and their partners at 11 and 10 timepoints, respectively. Table 2, Table 3 present a complete list of these data collection timepoints which correspond to the focal child’s average age within ALSPAC, as well as the ALSPAC data file and variable names. Nine of the timepoints are the same in both mothers and partners with respect to the focal child’s age: 18 weeks’ gestation, 8 weeks’ post-partum, 8 months, 1 year 9 months, 2 years 9 months, 5 years 1 month, 6 years 1 month, 8 years 1 month, and 11 years 2 months.

Due to attrition and other sources of missing data, sample size of mothers with EPDS data at each timepoint varies, from 12,151 at 18 weeks’ gestation to 4,107 at 18 years post-partum. In partners, sample sizes vary from 9,846 at 18 weeks’ gestation to 1,951 at 21 years post-partum ( Table 4, Table 5). A total of 14,169 mothers have valid EPDS data for at least one timepoint, with 2,854 of these having EPDS data for all 11 timepoints, and 1,476 mothers have data for zero EPDS assessments. In partners, 14,915 have at least one EPDS assessment, with 4,067 having all 10 assessments and 1,476 having zero. As can be seen in Tables 6- 7, missingness on EPDS data appears to be related to several demographic characteristics.

**Table 5.** Mother demographic characteristics by number of EPDS measurements in ALSPAC.

	No EPDS	One to ten EPDS	All eleven EPDS
**Maternal Education at 32 wks. gestation (n = 12,483)**			
O Level or lower	92.86%	69.68%	47.88%
A Level or Higher	7.14%	30.32%	52.12%
**Maternal Social Class at 18 wks. gestation (n = 11,117)**			
Skilled non-manual or lower	71.43%	71.85%	57.45%
Professional/Managerial/Technical	28.57%	28.15%	42.55%
**Mother Parity (n = 13,114)**			
First Born	56.58%	43.82%	47.80%
Second Born	19.74%	34.58%	36.68%
Third + Born	23.68%	21.59%	15.52%
**Maternal Age at child’s birth (n = 14,065)**			
< 25 Years	42.18%	27.02%	9.74%
25-29	34.08%	39.06%	37.67%
30-34	16.20%	25.13%	38.09%
35+	7.54%	8.80%	14.51%

**Table 6.** Partner demographic characteristics by number of EPDS measurements in ALSPAC.

	No EPDS	One to nine EPDS	All ten EPDS
**Partner Education at 32 wks. gestation (n = 12,001)**			
O Level or lower	30.35%	54.65%	72.91%
A Level or Higher	69.65%	45.65%	27.09%
**Partner Social Class at 32 wks. gestation (n = 10,378)**			
Professional/Managerial/Technical	64.64%	39.15%	31.73%
Skilled non-manual or lower	35.36%	60.85%	68.27%
**Partner Parity at 12 wks. gestation (n = 8,736)**			
First Born	46.78%	47.22%	46.50%
Second + Born	53.22%	52.78%	53.50%
**Partner Age at 18 wks. gestation (n = 9,652)**			
< 25 Years	1.23%	13.89%	30.95%
25-29	25.00%	35.22%	23.81%
30-34	43.17%	31.59%	33.33%
35+	30.60%	19.29%	11.90%

**Table 7.** Edinburgh Postnatal Depression Scale (EPDS) questions.

		Responses
1	I have been able to laugh and see the funny side of things	As much as I always could; Not quite so much now; Definitely not so much now; Not at all
2	I have looked forward with enjoyment to things	As much as I ever did; Rather less than I used to; Definitely less than I used to; Hardly at all
3	I have blamed myself unnecessarily when things went wrong	Yes, most of the time; Yes, some of the time; Not very often; No never
4	I have been anxious or worried for no good reason	No, not at all; Hardly ever; Yes, sometimes; Yes, often
5	I have felt scared or panicky for no very good reason	Yes, quite a lot; Yes, sometimes; No, not much; No, not at all
6	Things have been getting on top of me	Yes, most of the time; Yes, sometimes; No, hardly ever; No, not at all
7	I have been so unhappy that I have had difficulty sleeping	Yes, most of the time; Yes, sometimes; Not very often; No, not all
8	I have felt sad or miserable	Yes, most of the time; Yes, quite often; Not very often; No, not at all
9	I have been so unhappy that I have been crying	Yes, most of the time; Yes, quite often; Only occasionally; No, never
10	The thought of harming myself has occurred to me	Yes, quite often; Sometimes; Hardly ever; Never

### The Edinburgh Postnatal Depression Scale (EPDS)

The 10-item Edinburgh Postnatal Depression Scale (EPDS) ^11^ assesses depressive symptoms during the prior week. Respondents rate the frequency of each symptom on a Likert-type scale with four response options, the exact wording of which varies depending on the item. See Table 7 for a complete item list with corresponding response options. Responses from all ten questions are coded 0–3 and three items (1, 2, and 4) are reverse coded. All ten items are then summed to give a score ranging between 0 and 30, where higher scores indicate more depressive symptoms. The EPDS does not assess or provide information on the duration or intensity of depressive symptoms ^12^.

A score of 13 or higher on the EPDS is often used to indicate probable depression ^11, 37^. Others have used cut-off points of 12 ^38, 39^ and 10 ^17^. For simplicity, we present descriptive statistics using 10 and 13 as cut-offs. Syntax for creating the scores is available upon request.

### Characteristics of the EPDS in ALSPAC

The mean number of EPDS depressive symptoms and the prevalence of probable depression using both the 13 and 10 or greater cut-offs for mothers and partners are presented in Table 3, Table 4. In mothers, the greatest prevalence of depression occurs at 18 years post-partum, while the lowest is at 8 months’ post-partum ( Table 3). In partners, the greatest proportion with probable depression occurs at 21 years post-partum, while the lowest proportion is at 8 months’ post-partum ( Table 4).

Correlations of depressive symptoms between timepoints within mothers and partners are moderate (0.30 to 0.49) to large (< 0.50) ( Table 8, Table 9), providing initial evidence for predictive validity. Correlations between mother and partner depressive symptoms at the same timepoints are small to moderate (0.228-0.294) ( Table 10). Data users modelling depressive symptoms over time should use statistical methods to account for these correlations. Internal reliability of the EPDS at each timepoint for both mothers and partners in ALSPAC is good ( Table 3, Table 4).

**Table 8.** Table of correlations between EPDS scores on all occasions in mothers in ALSPAC.

	18 wks. gestation	32 wks. gestation	8 wks. post-partum	8 mos.	1 yr. 9 mos.	2 yrs. 9 mos.	5 yrs. 1 mo.	6 yrs. 1 mo.	8 yrs. 1 mo.	11 yrs. 2 mos.	18 yrs.
32 wks. gestation	0.643	-									
8 wks. post-partum	0.529	0.573	-								
8 mos.	0.502	0.549	0.614	-							
1 yr. 9 mos.	0.488	0.520	0.558	0.615	-						
2 yrs. 9 mos.	0.471	0.495	0.515	0.577	0.619	-					
5 yrs. 1 mo.	0.436	0.460	0.459	0.499	0.540	0.581	-				
6 yrs. 1 mo.	0.419	0.448	0.458	0.495	0.514	0.550	0.628	-			
8 yrs. 1 mo.	0.405	0.424	0.417	0.451	0.500	0.517	0.570	0.579	-		
11 yrs. 2 mos.	0.402	0.407	0.414	0.437	0.477	0.492	0.514	0.513	0.552	-	
18 yrs.	0.362	0.382	0.397	0.403	0.435	0.435	0.472	0.454	0.465	0.490	-

**Table 9.** Table of correlations between EPDS scores on all occasions in partners in ALSPAC.

	18 wks. gestation	8 wks. post- partum	8 mos.	1 yr. 9 mos.	2 yrs. 9 mos.	5 yrs. 1 mo.	6 yrs. 1 mo.	8 yrs. 1 mo.	11 yrs. 2 mos.	20 yrs.
8 wks. post-partum	0.578	-								
8 mos.	0.555	0.591	-							
1 yr. 9 mos.	0.520	0.554	0.584	-						
2 yrs. 9 mos.	0.472	0.493	0.526	0.605	-					
5 yrs. 1 mo.	0.422	0.461	0.471	0.514	0.537	-				
6 yrs. 1 mo.	0.445	0.460	0.479	0.496	0.491	0.631	-			
8 yrs. 1 mo.	0.407	0.419	0.441	0.455	0.462	0.513	0.567	-		
11 yrs. 2 mos.	0.390	0.418	0.403	0.447	0.423	0.523	0.532	0.542	-	
20 yrs.	0.368	0.377	0.368	0.378	0.347	0.477	0.430	0.429	0.493	-

**Table 10.** Table of correlations between EPDS scores on all nine common occasions in mothers and partners in ALSPAC.

	Mother at 18 wks. gestation	Mother at 8 wks. post- partum	Mother at 8 mos.	Mother at 1 yr. 9 mos.	Mother at 2 yrs. 9 mos.	Mother at 5 yrs. 1 mo.	Mother at 6 yrs. 1 mo.	Mother at 8 yrs. 1 mo.	Mother at 11 yrs. 2 mos.
Partner at 18 wks. gestation	0.249	0.192	0.175	0.175	0.161	0.140	0.131	0.122	0.118
Partner at 8 wks. post-partum	0.190	0.268	0.213	0.191	0.181	0.153	0.154	0.134	0.124
Partner at 8 mos.	0.218	0.221	0.280	0.220	0.202	0.182	0.180	0.150	0.131
Partner at 1 yr. 9 mos.	0.209	0.224	0.237	0.294	0.236	0.185	0.180	0.164	0.160
Partner at 2 yrs. 9 mos.	0.188	0.194	0.206	0.227	0.263	0.181	0.174	0.152	0.154
Partner at 5 yrs. 1 mo.	0.176	0.170	0.165	0.191	0.210	0.261	0.214	0.187	0.175
Partner at 6 yrs. 1 mo.	0.167	0.189	0.204	0.213	0.198	0.208	0.272	0.193	0.198
Partner at 8 yrs. 1 mo.	0.131	0.134	0.130	0.179	0.139	0.155	0.180	0.228	0.198
Partner at 11 yrs. 2 mos.	0.133	0.131	0.133	0.166	0.150	0.140	0.159	0.137	0.235

**Figure 1.**
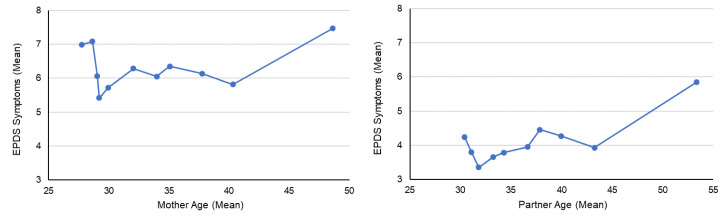
Mean number of EPDS depressive symptoms in mothers and partners according to average participant age.

**Figure 2.**
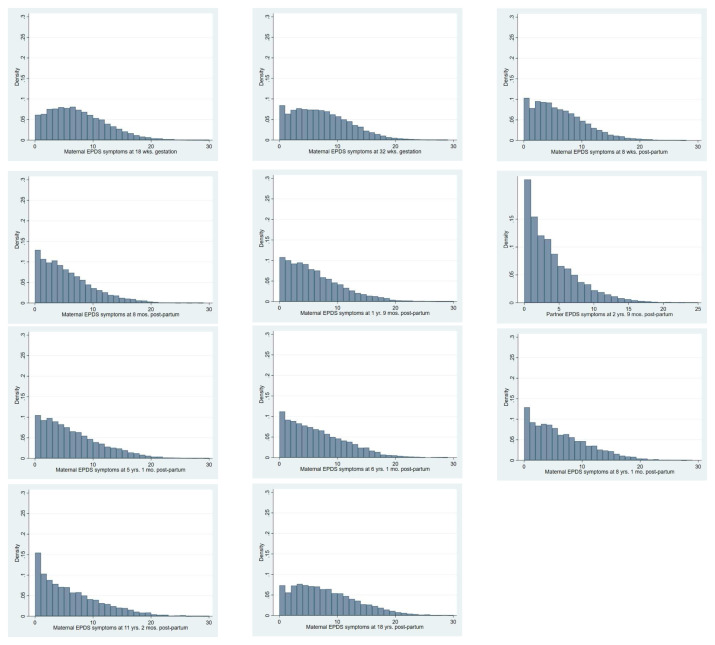
Histograms for the number of Edinburgh Postnatal Depression Scale (EPDS) symptoms in mothers at each of the eleven occasions in ALSPAC.

**Figure 3.**
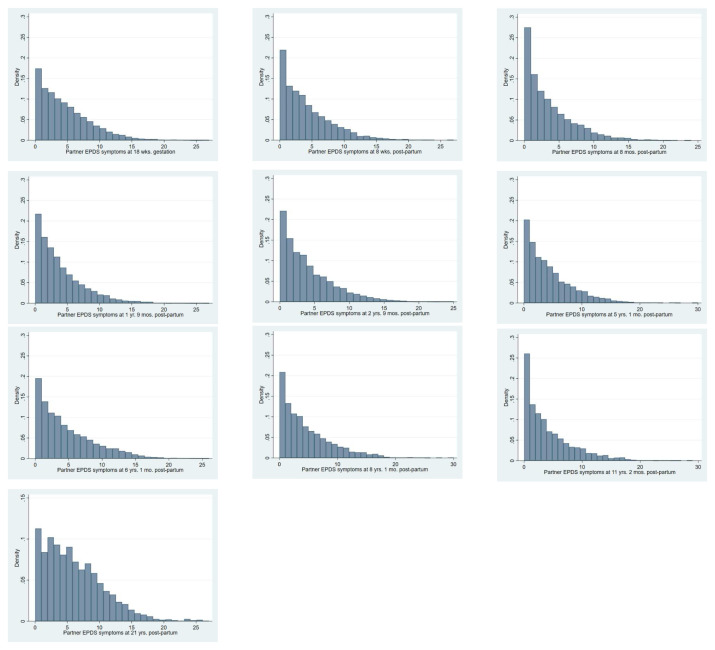
Histograms for the number of Edinburgh Postnatal Depression Scale (EPDS) symptoms in partners at each of the ten occasions in ALSPAC.

As can be seen in Tables 4 and 5, mothers’ depressive symptoms and rates of probable depression are consistently higher than their partners’. This is congruent with other research on gender differences in depression ^40^. Figure 1 provides a visual representation of the differences in mothers and partners in the average number of depressive symptoms over time. The mean number of depressive symptoms are also presented in histograms at each timepoint for mothers ( Figure 2) and partners ( Figure 3).

**Figure 4.**
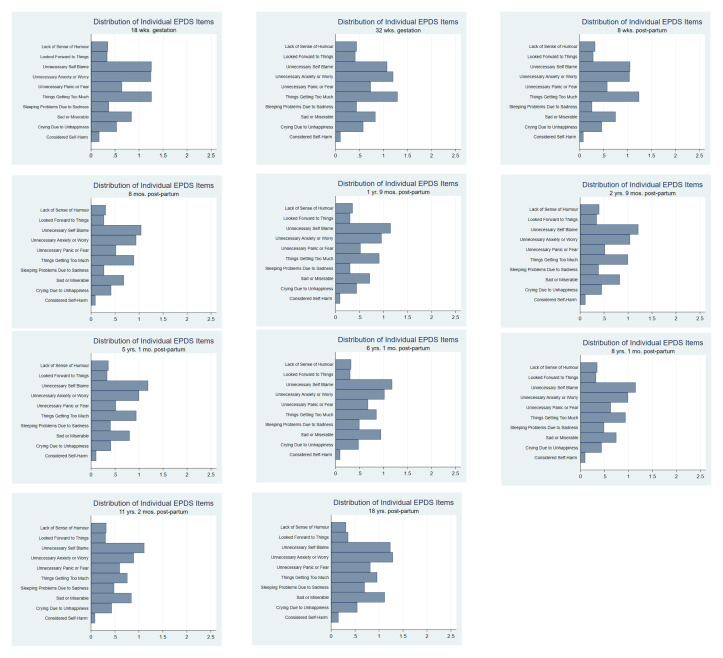
Means (scale of 0–3) of each specific Edinburgh Postnatal Depression Scale (EPDS) symptom at each of the eleven occasions in ALSPAC mothers.

**Figure 5.**
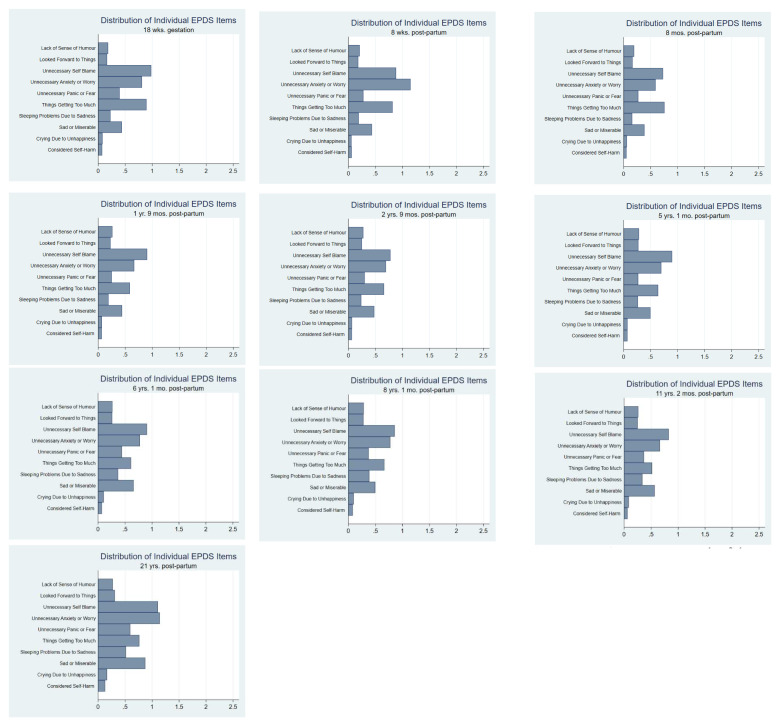
Means (scale of 0–3) of each specific Edinburgh Postnatal Depression Scale (EPDS) symptom at each of the eleven occasions in ALSPAC partners.

To help further illustrate these gender differences, Figure 4, Figure 5 present the distribution of the means (on a scale of 0–3) for each specific EPDS item in mothers and partners at all timepoints. In both mothers and partners, a common depressive symptom appears to be unnecessary self-blame. In mothers, being sad/miserable and anxious/worried are common symptoms, while in fathers, anxiety/worry and feeling overwhelmed (“things getting too much”) are common.

Some researchers have investigated whether the EPDS total score can be disaggregated into meaningful subscales ^41^. Using the ALSPAC data, Coates and colleagues ^42^ identified a three-factor solution to fit maternal EPDS data at 18 and 32 weeks’ gestation and 8 weeks and 8 months post-partum. The three factors were anhedonia (EPDS items 1 and 2), anxiety (EPDS items 3–6), and depression (EPDS items 7-10), similar to what others have found ^42, 43^.

**Figure 6.**
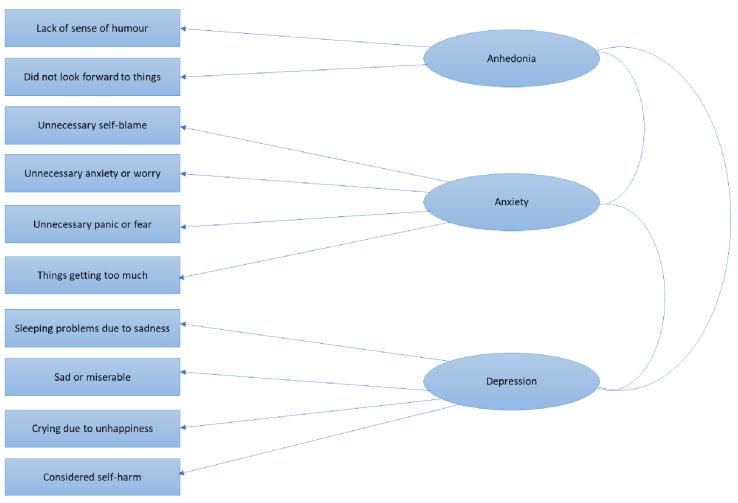
Factor structure of Edinburgh Postnatal Depression Scale (EPDS) symptom sub-scales.

We examined the fit of these three subscales using EPDS data in ALSPAC ( Figure 6) for mothers and partners at 18 weeks’ gestation and 11 years 2 months post-partum. Model fit statistics indicated generally good fit at both timepoints in both mothers and partners ( Table 11). Correlations between the three subscales within and between parents at 18 weeks’ gestation are presented in Table 12. Associations were strongest within rather than between parents.

**Table 11.** Model fit statistics for EPDS sub-scales in mothers and partners in ALSPAC.

	Mothers			Partners		
	RMSEA (90% CI)	CFI	TLI	RMSEA (95% CI)	CFI	TLI
18 wks. gestation (T1)	0.060 (0.057-0.062)	0.966	0.952	0.059 (0.056-0.062)	0.959	0.943
11 years 2 mos. post-partum (T9 partners T10 for mums)	0.076 (0.072-0.079)	0.961	0.946	0.067 (0.063-0.072)	0.961	0.946

CFI= comparative fit index; TLI= Tucker–Lewis index; RMSEA= root mean square error of approximation.

**Table 12.** Correlations between EPDS sub-scales in mothers and partners in ALSPAC at 18 wks. gestation.

	Mother Anhedonia	Mother Anxiety	Mother Depression	Partner Anhedonia	Partner Anxiety	Partner Depression
Mother Anhedonia	-					
Mother Anxiety	0.428	-				
Mother Depression	0.509	0.630	-			
Partner Anhedonia	0.161	0.141	0.168	-		
Partner Anxiety	0.141	0.197	0.185	0.383	-	
Partner Depression	0.142	0.161	0.219	0.461	0.564	-

### Related constructs in ALSPAC

Researchers using the EPDS in ALSPAC may want to take advantage of the wealth of information collected on related constructs. In addition to EPDS data in partners at 10 timepoints and in offspring, measures of anxiety, personality, nutrition, IQ, and biological characteristics such as genetic markers are available in ALSPAC. Users of ALSPAC data may consult the searchable database for additional constructs which may be of interest.

### ALSPAC publications using the EPDS

Over a dozen peer-reviewed papers that have used the EPDS in ALSPAC have now been published ^2, 4, 8, 10, 18, 24, 28, 30, 44– 48^). Interested readers can browse ALSPAC’s catalogue of over 2,000 current and published research papers on the study website.

### Considerations for the data

Like all longitudinal studies, missing data due to attrition must be taken into consideration when using ALSPAC data. However, due to the rich nature of information related to missingness in ALSPAC, users will be able to deal with missing data in a competent manner. Table 5, Table 6 list several of the potential characteristics which users can use to account for missing EPDS data. Users should take steps to handle missing data appropriately.

### Strengths and limitations of the data

A major strength of the ALSPAC data is the number of participants and that it is a general population sample. Another strength is the number of time points at which the entire EPDS was administered, thus providing identical assessments over time, and that it was administered not only to mothers but also to their partners. ALSPAC is therefore a rich resource for further exploration of questions related to gender differences in the presentation of depression. One possibility for future research with the ALSPAC data is to investigate whether there are gender differences in the depression symptom profiles of men and women ^49^. Some research indicates that when men are classified as being depressed by including symptoms such as risk-taking behaviour, anger attacks, and substance abuse, they are just as likely to meet criteria for depression as women ^50^.

A third advantage is that the offspring of the original cohort are now starting to have their own children, and these mothers, as well as their partners, are also being administered the EPDS. Two generations of EPDS data in mothers and partners are therefore available, enabling researchers to answer important questions about intergenerational depression. The data can also be linked to the wide range of other information already collected on mothers, partners and their children.

One limitation of the data is that the EPDS was not designed to measure information on the duration or intensity of depressive symptoms ^12^. A further limitation is the lack of consistency in the timing of measurement in mother and partner depressive symptoms in late childhood and adolescence. Both mothers and partners were administered the EPDS at child age 11 years 2 months, but the EPDS was not subsequently given to mothers until child age 18 and to partners at child age 21. The timing of these assessments will therefore somewhat limit questions which can be asked regarding depression in parents of adolescents. Another limitation of the ALSPAC data concerns racial and ethnic diversity. There are insufficient numbers of non-white participants to enable sub-group analyses.

## Data availability

### Underlying data

ALSPAC data are available through a system of managed open access. The application steps for ALSPAC data access are highlighted below.

1. Please read the ALSPAC access policy which describes the process of accessing the data in detail, and outlines the costs associated with doing so.

2. You may also find it useful to browse the fully searchable research proposals database, which lists all research projects that have been approved since April 2011.

3. Please submit your research proposal for consideration by the ALSPAC Executive Committee. You will receive a response within 10 working days to advise you whether your proposal has been approved. If you have any questions about accessing data, please email alspac-data@bristol.ac.uk.
