# Supplementary material for: Depressive symptoms measured using the Edinburgh Postnatal Depression Scale in mothers and partners in the ALSPAC Study: A data note
Source: Wellcome Open Res. 2020 Aug 25;5:108. Originally published 2020 May 29. [Version 2] doi: 10.12688/wellcomeopenres.15925.2 (PMC7385546; doi:10.12688/wellcomeopenres.15925.2)
Supplement: Peer review history

# Peer review: Reviewer response for version 1

**DOI:** 10.21956/wellcomeopenres.17467.r39486

**Published:** 2020-07-27

**Authors:** Sarah K. G. Jensen

**Affiliations:** 1 Boston College School of Social Work, Chestnut Hill, MA, USA

This is a well-written and thorough data note that will be helpful for a researcher who wishes to make use of the rich data on maternal and paternal depression within ALSPAC.

 Here are a few suggestions:

- Table 1: Consider adding the scores to the response categories.
- Description of the population section: I would move tables 6 and 7 to the start of the section. These tables are very relevant to someone who is determining whether or not this dataset can be used to explore their hypothesis. More information about partners may be helpful. E.g., were all partners male?
- Tables 2 and 3 are very detailed and only relevant to someone who has access to the data set. These do not have to be included here, but may instead be provided as an appendix.
- Figure 2 and 3 are very detailed and maybe not necessary. These response patterns are to be expected and can be described briefly in the text.
- I find the unit in Figures 4 and 5 somewhat confusing. How about the number of mothers/ partners who reported each symptom?
- It is fine to mention that factor analysis has been conducted to explore subscales/ dimensions within the EPDS. ALSPAC given the many waves of assessment provides an excellent opportunity to explore whether these data patterns consist over time, which could be informative but possibly too much to include here.
- When mentioning the CFA, it may also be relevant to mention Dr. Ted Barker’s work characterizing longitudinal latent classes (LLC) of maternal depression from pregnancy to 33 months (Barker, 2013 ^1^) in ALSPAC. He identified three classes of mothers, namely low risk, medium risk, possible clinical depression (10%).
- It may not be feasible, but it would be helpful to list all previous publications that have used EPDS in ALSPAC.

Are sufficient details of methods and materials provided to allow replication by others?

Yes

Is the rationale for creating the dataset(s) clearly described?

Yes

Are the datasets clearly presented in a useable and accessible format?

Yes

Are the protocols appropriate and is the work technically sound?

Yes

Reviewer Expertise:

Effects of early psychosocial experiences (including maternal depression) on early child development

I confirm that I have read this submission and believe that I have an appropriate level of expertise to confirm that it is of an acceptable scientific standard.

## References

[1] : The duration and timing of maternal depression as a moderator of the relationship between dependent interpersonal stress, contextual risk and early child dysregulation. *Psychol Med*.2013;43(8) : 10.1017/S0033291712002450 1587-96 10.1017/S0033291712002450 23127350PMC4104613

# Response

**Published:** 2020-08-17

**Authors:** Elise Paul

**Affiliations:** University of Bristol, UK

We thank you for taking the time to review our data note. See our responses below. 

 Table 1: Consider adding the scores to the response categories.

- We are not sure what you mean here. As in, the mean scores at each time point for each of the item? If so, we believe this would provide readers with an unnecessary amount of information.

Description of the population section: I would move tables 6 and 7 to the start of the section. These tables are very relevant to someone who is determining whether or not this dataset can be used to explore their hypothesis. More information about partners may be helpful. E.g., were all partners male?

- Tables 6 and 7 are now moved up in the section. All partners were indeed male, and this point has been clarified.

Tables 2 and 3 are very detailed and only relevant to someone who has access to the data set. These do not have to be included here but may instead be provided as an appendix.

- We agree with this point, but unfortunately appendices are not possible with Wellcome data notes.

Figures 2 and 3 are very detailed and maybe not necessary. These response patterns are to be expected and can be described briefly in the text.

- We have decided to keep these, as some readers may like to see visual representations of symptom patterns. 

I find the unit in Figures 4 and 5 somewhat confusing. How about the number of mothers/ partners who reported each symptom?

- We agree that this needed clarifying. Rather than dichotomising answers, we present the mean on a 0-3 scale for each item and have clarified this in the text.

It is fine to mention that factor analysis has been conducted to explore subscales/ dimensions within the EPDS. ALSPAC given the many waves of assessment provides an excellent opportunity to explore whether these data patterns consist over time, which could be informative but possibly too much to include here. 

 When mentioning the CFA, it may also be relevant to mention Dr. Ted Barker’s work characterizing longitudinal latent classes (LLC) of maternal depression from pregnancy to 33 months (Barker, 20131) in ALSPAC. He identified three classes of mothers, namely low risk, medium risk, possible clinical depression (10%).

- We appreciate this suggestion and citation but feel it would be beyond the scope of this data note to include this.

It may not be feasible, but it would be helpful to list all previous publications that have used EPDS in ALSPAC.

- This is an excellent idea. We have cited more previous publications and directed readers to the ALSPAC publications database.

# Peer review: Reviewer response for version 1

**DOI:** 10.21956/wellcomeopenres.17467.r38891

**Published:** 2020-06-01

**Authors:** Karen Thorpe

**Affiliations:** 1 Institute for Social Science Research, The University of Queensland, Indooroopilly, Queensland, Australia

The report gives a thorough account of the ALSPAC data and provides potential users with a clear outline of the available data, potential uses, strengths and limitations.

 The authors might reference the significant background work undertaken to ensure. The EPDS was suitable for use with partners and for use outside the postpartum period (citations below) ^1^, ^2^, ^3^, ^4^

 There are some typographical errors - notable in the abstract. "with' should be "when". Some minor errors in the reference list.

Are sufficient details of methods and materials provided to allow replication by others?

Yes

Is the rationale for creating the dataset(s) clearly described?

Yes

Are the datasets clearly presented in a useable and accessible format?

Yes

Are the protocols appropriate and is the work technically sound?

Yes

Reviewer Expertise:

Child Psychology, Developmental Science, Longitidinal research, Life course studies

I confirm that I have read this submission and believe that I have an appropriate level of expertise to confirm that it is of an acceptable scientific standard.

## References

[1] : The effects of psychosocial factors on the mother's emotional well-being during early parenthood: A cross-cultural study of britain and greece. *Journal of Reproductive and Infant Psychology*.1992;10(4) : 10.1080/02646839208403954 205-217 10.1080/02646839208403954

[2] : The effects of psychosocial factors on the emotional well-being of women during pregnancy: A cross-cultural study of britain and greece. *Journal of Reproductive and Infant Psychology*.1992;10(4) : 10.1080/02646839208403953 191-204 10.1080/02646839208403953

[3] : Changes in depression during and following pregnancy. ALSPAC Study Team. Study of Pregnancy and Children. *Paediatr Perinat Epidemiol*.1996;10(3) : 10.1111/j.1365-3016.1996.tb00051.x 279-93 10.1111/j.1365-3016.1996.tb00051.x 8822771

[4] : A study of the use of the Edinburgh postnatal depression scale with parent groups outside the postpartum period. *Journal of Reproductive and Infant Psychology*.1993;11(2) : 10.1080/02646839308403204 119-125 10.1080/02646839308403204

# Response

**Published:** 2020-06-03

**Authors:** Elise Paul

**Affiliations:** University of Bristol, UK

Thank you very much for taking the time to review our data note. We have taken the typographical errors into account.

# Response

**Published:** 2020-08-17

**Authors:** Elise Paul

**Affiliations:** University of Bristol, UK

We thank you for taking the time to review our data note. We have taken your suggestion to add more references on background work using the EPDS outside of the postpartum period. We have also fixed the typographical errors.
